# Supplementary material for: The genomic sequence of Exiguobacterium chiriqhucha str. N139 reveals a species that thrives in cold waters and extreme environmental conditions
Source: PeerJ. 2017 Apr 19;5:e3162. doi: 10.7717/peerj.3162 (PMC5399880; doi:10.7717/peerj.3162)
Supplement: Supplemental Information 1 [file peerj-05-3162-s001.doc]

**Supplemental Material**

*Exiguobacterium chiriqhucha* str. N139, a halotolerant, UV-B and metal resistant bacteria from a high-altitude Andean lake

Ana Gutiérrez-Preciado^1†‡^, Carlos Vargas-Chávez^1†^, Mariana Reyes-Prieto^1^, Omar F Ordoñez^2^, Diego Santos-Garcia^1^, Tania Rosas-Pérez^1^, Jorge Valdivia-Anistro^3^, Eria A Rebollar^4^, Andrés Saralegui^5^, Andrés Moya^1^, Enrique Merino^6^, María Eugenia Farias^2^, Amparo Latorre^1*^ and Valeria Souza^3*^.

^*^Corresponding authors: Amparo Latorre E-mail: amparo.latorre@uv.es; Valeria Souza E-mail: souza@servidor.unam.mx

^†^Equal contributors

^1^Unidad de Genética Evolutiva, Instituto Cavanilles de Biodiversidad y Biología Evolutiva, Universidad de Valencia, Calle Catedrático José Beltrán Martínez 2, 46980, Paterna, Valencia, Spain

^2^Laboratorio de Investigaciones Microbiológicas de Lagunas Andinas (LIMLA), Planta Piloto de Procesos Industriales Microbiológicos (PROIMI), Consejo Nacional de Investigaciones Científicas y Técnicas (CONICET), Av. Belgrano y Pasaje Caseros, 4000 San Miguel de Tucumán, Argentina

^3^Departamento de Ecología Evolutiva, Instituto de Ecología, Universidad Nacional Autónoma de México, México D.F., México

^4^Department of Biology, James Madison University, Harrisonburg, Virginia, 22801, United States of America

^5^Laboratorio Nacional de Microscopía Avanzada, Instituto de Biotecnología, Universidad Nacional Autónoma de México, Apdo, Postal 510-3, Cuernavaca, Morelos 62250, México

^6^Departamento de Microbiología Molecular, Instituto de Biotecnología, Universidad Nacional Autónoma de México, Apdo, Postal 510-3, Cuernavaca, Morelos 62250, México

^‡^Present address: Ecologie Systématique Evolution, CNRS, Université Paris-Sud, AgroParisTech, Université Paris-Saclay, Orsay, France

Data deposition: This Whole Genome Shotgun project has been deposited at GenBank under the accession JMEH00000000. The version described in this paper is version JMEH001000000.1; BioSample SAMN02732272

Short title: Genome of *E. pavilionensis* str. N139

**Material and Methods**

*Number of copies of the rRNA operon and megaplasmids extraction*

The strains N139 and S17 of the Exiguobacterium genus were cultured aerobically under different media in agreement to their isolation conditions. The strains N139 and S17 were grown in Luria-Bertani broth. Both cultures were incubated overnight at 35 ºC with shaking at 150 rpm. Genomic DNA blocks and cellular digestion were performed following the protocol used by (Goering, 2010).

Megaplasmids were separated by Pulsed Field Gel Electrophoresis (PFGE) with the CHEF-DR II System (Bio-Rad). Electrophoresis was carried out in 1.2% of Seakem Gold agarose (BioWhittaker Molecular Applications) gel in 0.5X TBE buffer (Bio-Rad) at 11ºC. Electrophoresis conditions were divided in two stages. First, pulse time ramped from 0.47 s to 26.3s for 12 h at 4.5V cm^-1^ and in a second place, pulse time ramped from 20 s to 1.10 s for 16 h at 4.5V cm^-1^. DNA was extensively treated with proteinase K to remove any enzyme that could degrade DNA, as well as supercoiling associated topoisomerases. Hence, the bands in the gel represent extended, but circular megaplasmids.

Both analyses were made in duplicate; *Exiguobacterium chiriqhucha* str. N139 has three megaplasmids of sizes 48.5 kb, 144 kb and 244 kb. Two electrophoresis assays were performed, only the the second is presented as the buffer was complemented with 50 μM of thiourea in agreement to (Lee, Bussema & Schmidt, 2009) in order to increase the resolution in the electrophoresis, (Figure S2).

The determination of the number of copies of the rRNA operons was performed by endonuclease digestion and PFGE. Agarose blocks were equilibrated in 1X NEBuffer 4 (New England Biolabs) for 30 min at 4 ºC. Genomic DNA digestion was achieved with fresh 1X NEBuffer 4 containing 15U of I-*Ceu*I endonuclease, incubated overnight at 37 ºC. This enzyme specifically cleaves the 23S rDNA. Fragments were separated with the CHEF-DR II System (Bio-Rad). Electrophoresis was carried out in 1% of Seakem Gold agarose (BioWhittaker Molecular Applications) gel in 0.5X TBE buffer (Bio-Rad) at 11 ºC. Electrophoresis conditions were divided in two stages: *1)* Pulse time ramped from 6.75 s to 2 min for 20 h at 4V cm^-1^ and, *2)* Pulse time ramped from 0.22 s to 5.10 s for 15 h at 6V cm^-1^. Since the smaller fragments might be difficult to see, the rRNA operon quantification was confirmed by Southern hybridization with specific labeled DNA probes designed for the *Bacillus* genus. Labeled probes were designated using the 5' HV region of the 16S rDNA (275 bp; 70-344 position) and an internal region of the 23S rDNA gene (413 bp; 2283-2696 position) (Valdivia-Anistro et al., 2015). Agarose gels were radiated with UV light for one minute in a UV Cross-linker (UVP) for DNA fixation and washed in 250 mM HCl solution for 15 m with moderate shaking. Gels were damped with denaturing buffer (1.5 M NaCl, 0.5 M NaOH) for 2 h, and dipped in neutralization buffer (0.5 M Tris/HCl, 1.5 M NaCl; pH 8.0) for 2 h. Finally, the gels were transferred to an N^+^ nylon membrane (Amersham Biosciences) by upward capillary transfer. DNA labeling and hybridization were performed following the instructions of the *DIG High* Prime DNA Labeling and Detection Starter Kit II (Roche). As observed in Figure S1, *E. chiriqhucha* str. N139 has 10 ribosomal rRNA operons.

**Supplemental Figures**

**Supplemental Figure 1**. Megaplasmids in *Exiguobacterium chiriqhucha* str. N139 and sp. S17. 1) Weight molecular marker 50-100 kb; 2) N139; 3) S17; 4) Weight molecular marker 0.1-200 kb; 5) *E. coli* 3496. Three megaplasmids are observed in the *Exiguobacterium* strains from HAAL of sizes 250.57, 137.48 and 48 kb in *E. chiriqhucha.* str. N139; and 251.98, 140.8 and 47.94 kb in *E*. sp. S17, whilst in *E. coli* str. 3496 (Laboratorio de Evolución Molecular y Experimental, UNAM strain collection) only one megaplasmid of 536 kb was observed. The molecular weight of the plasmids was calculated using the software BioNumerics 7.


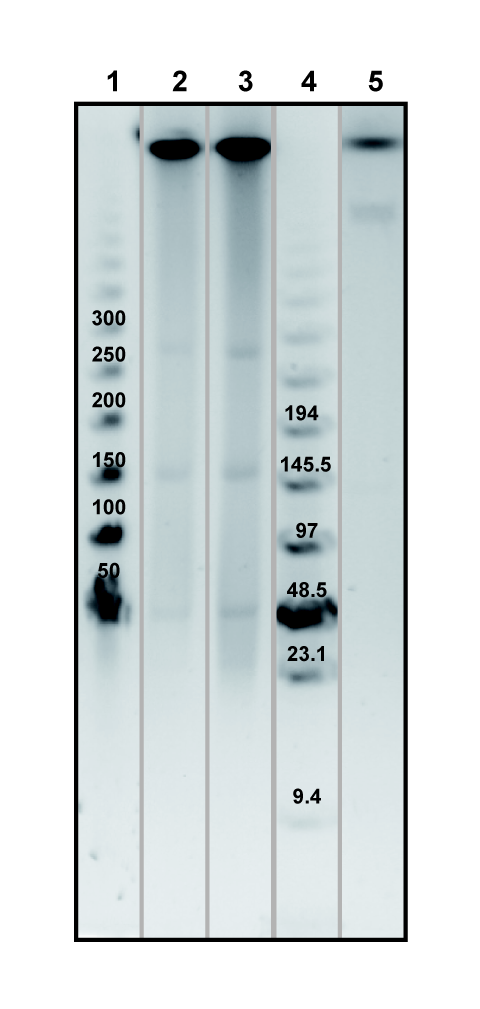


**Supplemental Figure 2.** rRNA copy numbers in *E. chiriqhucha* str. N139 and *E.* sp. S17*. r*ibosomal operons are shown in the gel, obtained by digesting the whole genome with enzyme I-*Ceu*l, as described in the Supplementary Methods. This enzyme binds to a 23 bp-sequence located in the middle of the 23S rRNA gene. The gel was exposed 0.5s to UV light. StLT2 corresponds to *Salmonella typhimurium* LT2 and it is used as the positive control, since it has 7 copies of the ribosomal operon (Liu, Hessel & Sanderson, 1993). Both *Exiguobacterium* strains from HAAL have 10 ribosomal operons, in agreement to what had been reported for *Exiguobacterium sibiricum* 255-15 (Rodrigues et al., 2006).


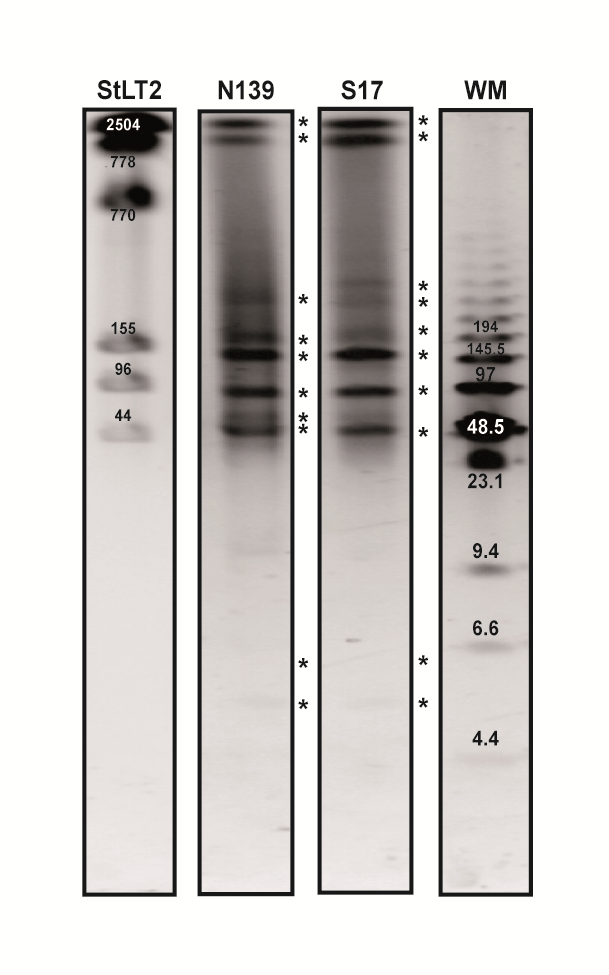


**Suplementary Figure 3**. Clade II Strain Specific Genes through their COG categories. The pangenome of all the complete genome sequences of the *Exiguobacterium* genus from clade II was calculated with the OrthoMCL pipeline (Li, Jr & Roos, 2003; Fischer et al., 2011). From the pangenome, the core genome was calculated, as well as the Strain Specific Genes (SSGs). These SSGs could confer unique capabilities to each of the *Exiguobacterium* that could favor their adaptability to the environment they deal with. COGs were predicted for 66% of the SSGs following the methodology described in the Material and Methods section. Functional profiles from this set of genes are represented in the heatmap. The Poorly Characterized proteins are overrepresented in the Exiguos. COG categories are as follows. For CELLULAR PROCESSES AND SIGNALING: [D] Cell cycle control, cell division, chromosome partitioning; [M] Cell wall/membrane/envelope biogenesis; [N] Cell motility; [O] Post-translational modification, protein turnover, and chaperones; [T] Signal transduction mechanisms; [U] Intracellular trafficking, secretion, and vesicular transport; [V] Defense mechanisms; [W] Extracellular structures; [Y] Nuclear structure; [Z] Cytoskeleton; INFORMATION STORAGE AND PROCESSING: [A] RNA processing and modification; [B] Chromatin structure and dynamics; [J] Translation, ribosomal structure and biogenesis; [K] Transcription; [L] Replication, recombination and repair; METABOLISM: [C] Energy production and conversion; [E] Amino acid transport and metabolism; [F] Nucleotide transport and metabolism; [G] Carbohydrate transport and metabolism; [H] Coenzyme transport and metabolism; [I] Lipid transport and metabolism; [P] Inorganic ion transport and metabolism; [Q] Secondary metabolites biosynthesis, transport, and catabolism; POORLY CHARACTERIZED: [R] General function prediction only; and [S] Function unknown.


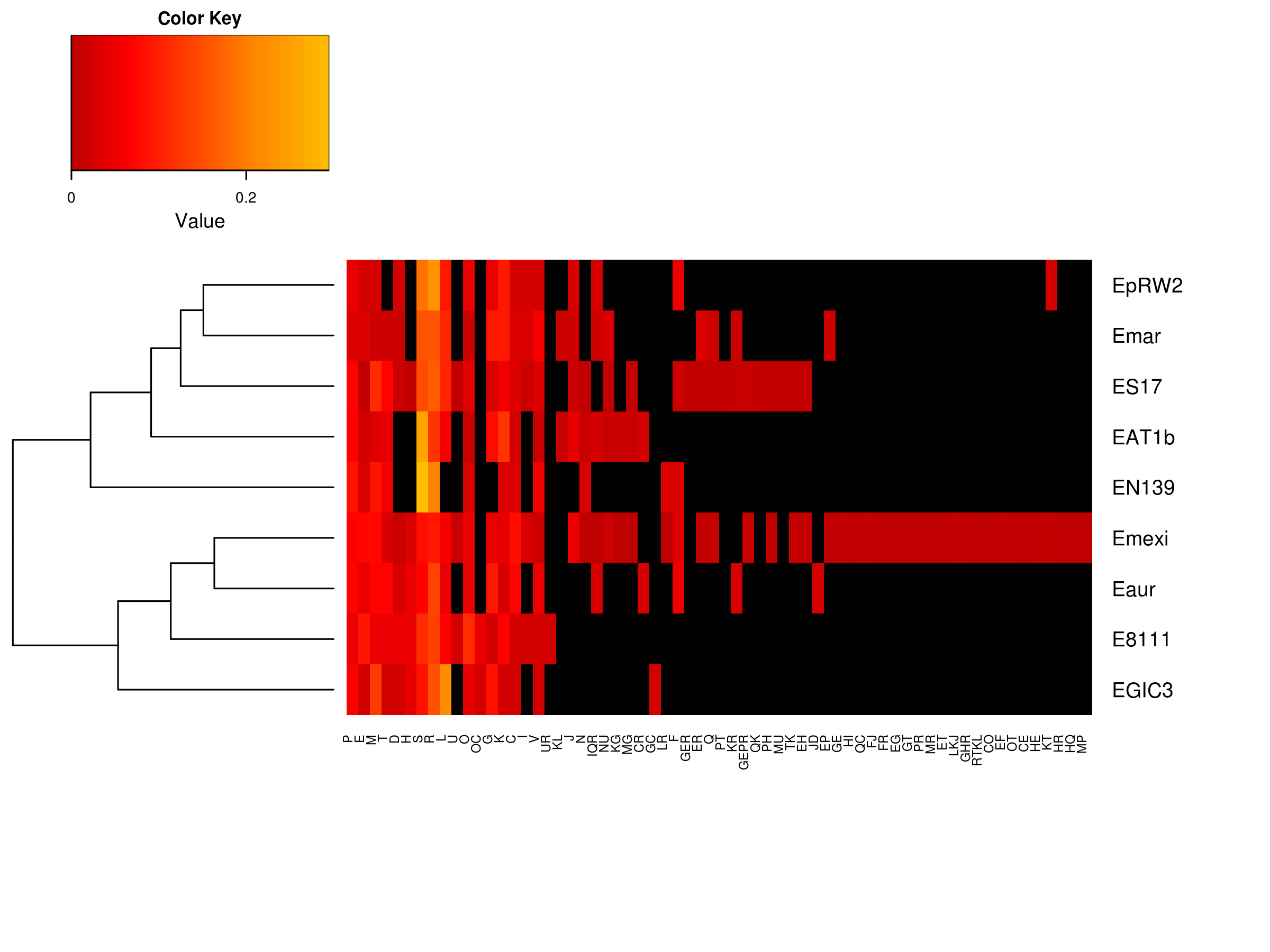


**Table S1**. Physiological characterization of *Exiguobacterium chiriqhucha* str. N139.

| **Carbon source** | *Exiguobacterium chiriqhucha* str. N139 |
| --- | --- |
| **β-Methylglucoside** | **YES** |
| Galactonic acid | NO |
| L-arginine | NO |
| Pyruvic acid | NO |
| Xylose | NO |
| **Galacturonic acid** | **YES** |
| **L-asparagine** | **YES** |
| **Tween 40** | **YES** |
| Erythritol | NO |
| 2-Hydroxybenzoic acid | NO |
| Phenylalanine | NO |
| Tween 80 | NO |
| Mannitol | NO |
| 4-Hydroxybenzoic acid | NO |
| **L-Serine** | **YES** |
| Cyclic Dextrin | NO |
| **N-acetyl-glucosamine** | **YES** |
| **Hydroxybutyric acid** | **YES** |
| L-Threonine | NO |
| Glucogen | NO |
| Glucosaminic acid | NO |
| **Itaconic acid** | **YES** |
| Glycyl-L-glutamic acid | NO |
| Cellobiose | NO |
| Glucose-1-P | NO |
| **Ketobutyric acid** | **YES** |
| Phenethylamine | NO |
| Lactose | NO |
| Gycerol phosphate | NO |
| Malic acid | NO |
| **Putrescine** | **YES** |

Assimilation of 31 single carbon sources was tested for the type strain *E. sibiricum* and *E. chiriqhucha* str. N139 using EcoPlates (Biolog Hayward, CA, USA) according to the manufacturer’s protocol. YES means that the strain grew by using a specific sole carbon source. Ten carbon sources were only positive for *E. chiriqhucha* str. N139.

***Table S2. ANI calculations for the E. chiriqhucha str. N139 genome against five strains of the Exiguobacterium genus.***

| ***E. chiriqhucha* str. N139 *vs…*** | ANI (2-ways) | AAI (2-ways) |
| --- | --- | --- |
| …*E. pavilionensis* RW2 | 97.57% | 97.67% |
| …*E.* GIC31 | 97.32% | 97.60% |
| …*E. mexicanum* | 90.34% | 94.48% |
| …*E. aurantiacum* | 83.66% | 87.05% |
| …*E.* S17 | 82.98% | 86.86% |

The ANI (Average Nucleotide Identity) uses both best hits (one-way ANI) and reciprocal best hits (two-way ANI) between two complete genome sequences to estimate an identity score, the ANI (Goris et al., 2007). Typically, the ANI values between genomes of the same species are above 95% (e.g., *E. coli*). Values below 75% are not to be trusted. The ANI calculations were performed with the ANI calculator (Goris et al., 2007). The AAI (Average Amino Acid Identity) is a similar metric but uses amino acids instead of nucleic acids, it is normally used for distantly related populations as it provides more resolution than the ANI score. Both scores are significant information when defining bacterial species (Rodriguez-R & Konstantinidis, 2014).

**Table S3.** Pangenome of the genus *Exiguobacterium* from clade II

| **Gene set** | Genes |
| --- | --- |
| Pangenome | 5267 |
| Disposable genome | 1487 |
| Core Genome | 2116 |
| Strain Specific (SS) | 1664 |
| SS *E. chiriqhucha* str. RW2 | 85 |
| SS *E. chiriqhucha* str. N139 | 59 |
| SS *E. chiriqhucha* str. GIC31 | 63 |
| SS *E.* AT1b | 183 |
| SS *E.* S17 | 392 |
| SS *E. aurantiacum* | 73 |
| SS *E. marinum* | 106 |
| SS *E. mexicanum* | 624 |
| SS *E.* 8-11-1 | 79 |

Statistics of the Pangenome from the genus *Exiguobacterium* from clade II. From the 1,664 Strain Specific genes, 1,102 could be assigned to COGs.

**References**

Fischer S., Brunk BP., Chen F., Gao X., Harb OS., Iodice JB., Shanmugam D., Roos DS., Stoeckert CJ. 2011. Using OrthoMCL to Assign Proteins to OrthoMCL-DB Groups or to Cluster Proteomes Into New Ortholog Groups. *Current protocols in bioinformatics editoral board Andreas D Baxevanis et al* Chapter 6:Unit6.12.

Goering R V. 2010. Pulsed field gel electrophoresis: A review of application and interpretation in the molecular epidemiology of infectious disease. *Infection, Genetics and Evolution* 10:866–875. DOI: 10.1016/j.meegid.2010.07.023.

Goris J., Konstantinidis KT., Klappenbach J a., Coenye T., Vandamme P., Tiedje JM. 2007. DNA-DNA hybridization values and their relationship to whole-genome sequence similarities. *International Journal of Systematic and Evolutionary Microbiology* 57:81–91. DOI: 10.1099/ijs.0.64483-0.

Lee ZMP., Bussema C., Schmidt TM. 2009. rrn DB: Documenting the number of rRNA and tRNA genes in bacteria and archaea. *Nucleic Acids Research* 37:489–493. DOI: 10.1093/nar/gkn689.

Li L., Jr CJS., Roos DS. 2003. OrthoMCL: Identification of Ortholog Groups for Eukaryotic Genomes. *Genome Research*. 2178–2189. DOI: 10.1101/gr.1224503.candidates.

Liu S., Hessel A., Sanderson KE. 1993. Genomic mapping with I-Ceu I, an intron-encoded endonuclease specific for genes for ribosomal RNA, in Salmonella spp ., Escherichia coli , and other bacteria. *Proceedings of the National Academy of Sciences* 90:6874–6878.

Rodrigues DF., Goris J., Vishnivetskaya T., Gilichinsky D., Thomashow MF., Tiedje JM. 2006. Characterization of Exiguobacterium isolates from the Siberian permafrost. Description of Exiguobacterium sibiricum sp. nov. *Extremophiles: Life Under Extreme Conditions* 10:285–294.

Rodriguez-R LM., Konstantinidis KT. 2014. Bypassing Cultivation to Identify Bacterial Specie*s. Micr*obe 9:111–118.

Valdivia-Anistro JA., Eguiarte-Fruns LE., Delgado-Sapién G., Márquez-Zacaríaz P., Gasca-Pineda J., Learned J., Elser JJ., Olmedo-Alvarez G., Souza V. 2015. Variability of rRNA operon copy number and growth rate dynamics of Bacillus isolated from an extremely oligotrophic aquatic ecosystem. *Frontiers in Microbiology* 6:1486. DOI: 10.3389/fmicb.2015.01486.
