# Supplementary figures and images for: The genomic sequence of Exiguobacterium chiriqhucha str. N139 reveals a species that thrives in cold waters and extreme environmental conditions
Source: PeerJ. 2017 Apr 19;5:e3162. doi: 10.7717/peerj.3162 (PMC5399880; doi:10.7717/peerj.3162)

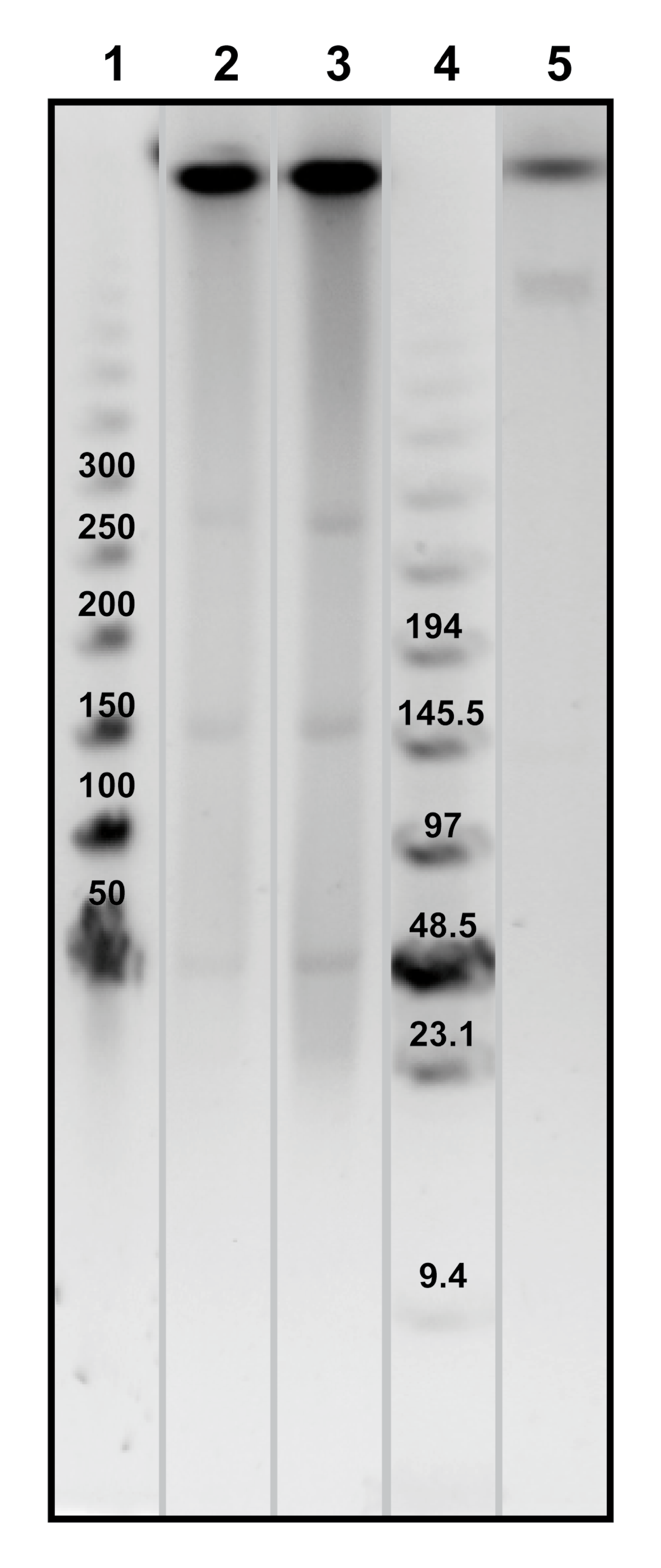

Supplement: Figure S1 — (1) Weight molecular marker 50–100 kb; (2) N139; (3) S17; (4) Weight molecular marker 0.1–200 kb; (5) E. coli 3496. Three megaplasmids are observed in the Exiguobacterium strains from HAAL of sizes 250. 57, 137.48 and 48 kb in E. chiriqhucha. str. N139; and 251.98, 140.8 and 47.94 kb in E. sp. S17, whilst in E. coli str. 3496 (Laboratorio de Evolución Molecular y Experimental, UNAM strain collection) only one megaplasmid of 536 kb was observed. The molecular weight of the plasmids was calculated using the software BioNumerics 7. [file peerj-05-3162-s002.png]

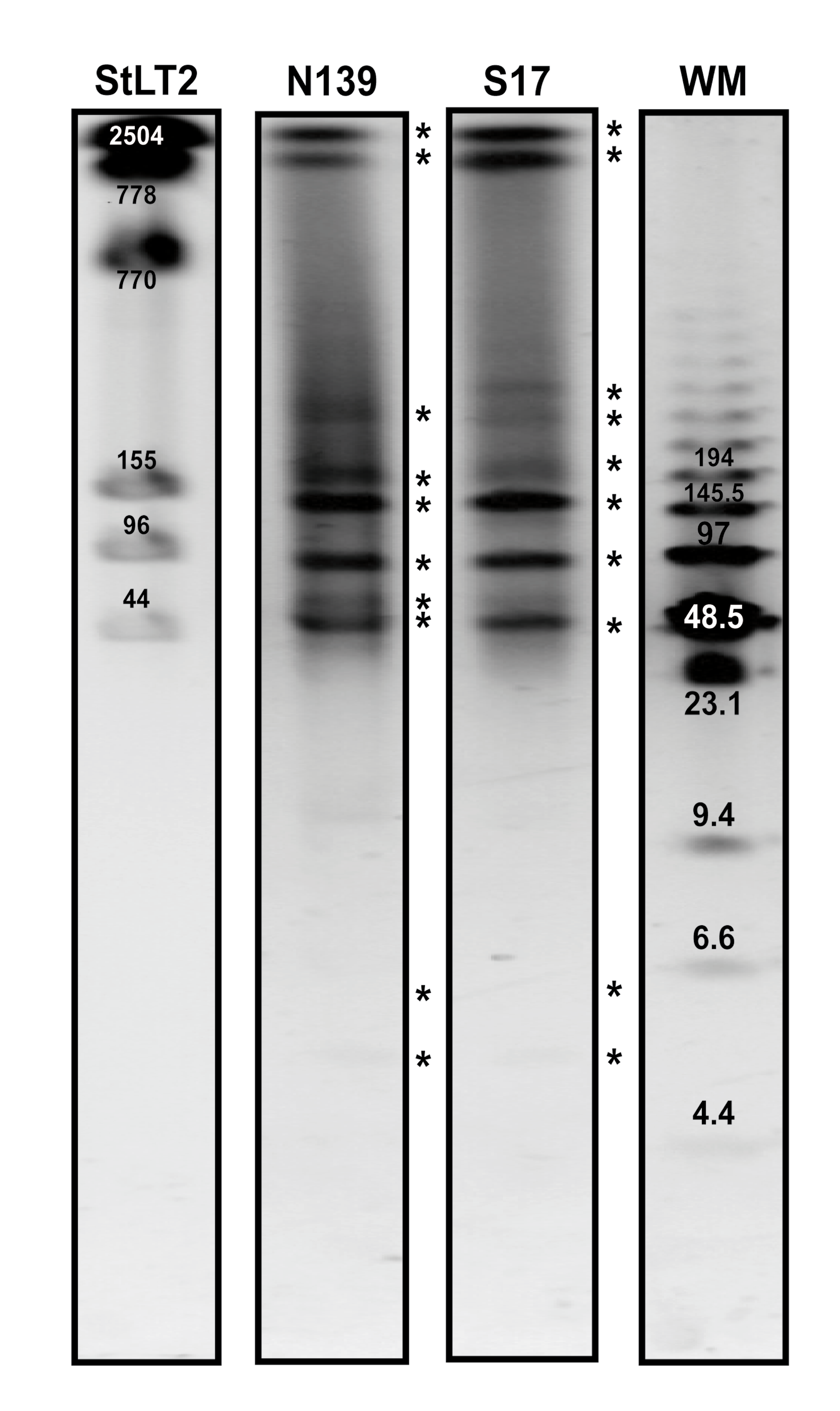

Supplement: Figure S2 — r ibosomal operons are shown in the gel, obtained by digesting the whole genome with enzyme I-Ceu l, as described in the Supplementary Methods. This enzyme binds to a 23 bp-sequence located in the middle of the 23S rRNA gene. The gel was exposed 0.5s to UV light. StLT2 corresponds to Salmonella typhimurium LT2 and it is used as the positive control, since it has 7 copies of the ribosomal operon (Liu, Hessel & Sanderson, 1993). Both Exiguobacterium strains from HAAL have 10 ribosomal operons, in agreement to what had been reported for Exiguobacterium sibiricum 255–15 (Rodrigues et al., 2006). [file peerj-05-3162-s003.png]

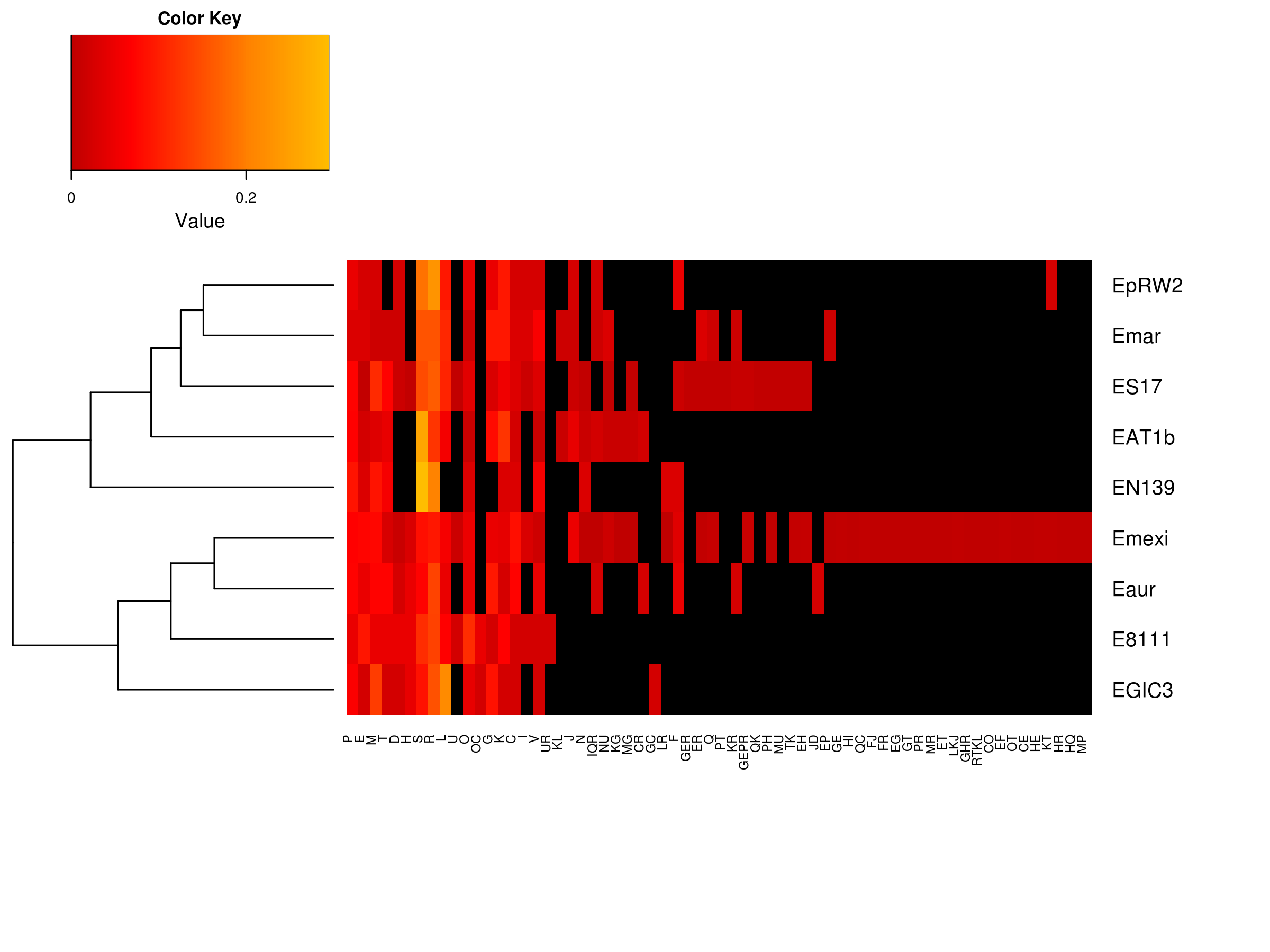

Supplement: Figure S3 — The pangenome of all the complete genome sequences of the Exiguobacterium genus from clade II was calculated with the OrthoMCL pipeline (Li, Jr & Roos, 2003; Fischer et al., 2011). From the pangenome, the core genome was calculated, as well as the Strain Specific Genes (SSGs). These SSGs could confer unique capabilities to each of the Exiguobacterium that could favor their adaptability to the environment they deal with. COGs were predicted for 66% of the SSGs following the methodology described in the Material and Methods section. Functional profiles from this set of genes are represented in the heatmap. The Poorly Characterized proteins are overrepresented in the Exiguos. COG categories are as follows. For CELLULAR PROCESSES AND SIGNALING: [D] Cell cycle control, cell division, chromosome partitioning; [M] Cell wall/membrane/envelope biogenesis; [N] Cell motility; [O] Post-translational modification, protein turnover, and chaperones; [T] Signal transduction mechanisms; [U] Intracellular trafficking, secretion, and vesicular transport; [V] Defense mechanisms; [W] Extracellular structures; [Y] Nuclear structure; [Z] Cytoskeleton; INFORMATION STORAGE AND PROCESSING: [A] RNA processing and modification; [B] Chromatin structure and dynamics; [J] Translation, ribosomal structure and biogenesis; [K] Transcription; [L] Replication, recombination and repair; METABOLISM: [C] Energy production and conversion; [E] Amino acid transport and metabolism; [F] Nucleotide transport and metabolism; [G] Carbohydrate transport and metabolism; [H] Coenzyme transport and metabolism; [I] Lipid transport and metabolism; [P] Inorganic ion transport and metabolism; [Q] Secondary metabolites biosynthesis, transport, and catabolism; POORLY CHARACTERIZED: [R] General function prediction only; and [S] Function unknown. [file peerj-05-3162-s004.png]
